# Diagnosis of Rheumatoid Vasculitis From Ischemic Change in Hands: A Case Report

**DOI:** 10.7759/cureus.48962

**Published:** 2023-11-17

**Authors:** Ryuichi Ohta, Chiaki Sano

**Affiliations:** 1 Communiy Care, Unnan City Hospital, Unnan, JPN; 2 Community Medicine Management, Shimane University Faculty of Medicine, Izumo, JPN

**Keywords:** early detection and intervention, community hospital management, polyarticular joint pains, clinical heterogeneity, hypocomplementemia, rituximab, anti-cd20 monoclonal antibody, ischemic hands, rheumatoid vasculitis, rheumatoid arthritis

## Abstract

Rheumatoid arthritis (RA) is a chronic inflammatory disorder with a wide clinical heterogeneity. Among its complications, rheumatoid vasculitis (RV) is notable for its severity and potential to involve multiple organ systems. A particularly serious manifestation of RV is ischemia, which is indicative of advanced vasculitic involvement and a significant risk of tissue damage. This case report describes an 83-year-old male with RA who presented with polyarticular joint pain and hand ischemia. Despite the initial diagnosis of RA exacerbation, worsening systemic symptoms without identifiable infectious causes and hypocomplementemia led to the diagnosis of RV exacerbation. Initial management with steroids showed temporary improvement. However, relapse after dose reduction prompted the administration of rituximab, an anti-cluster-of-differentiate-20 (anti-CD20) monoclonal antibody, which yielded favorable outcomes. This case underscores the importance of clinical vigilance in older patients with RA for signs, such as ischemic hands, emphasizing the pivotal role of early detection and intervention in RV management, particularly in community hospital settings.

## Introduction

Rheumatoid arthritis (RA) is a chronic inflammatory disorder that primarily affects the synovial joints, often leading to systemic manifestations [1]. Its multifactorial etiology and clinical heterogeneity challenge both diagnosis and management [1]. Within the spectrum of complications associated with RA, rheumatoid vasculitis (RV) emerges as one of the most severe [2]. As a systemic vasculitis, RV can involve multiple organ systems and manifests in diverse clinical presentations, making early recognition pivotal for preventing adverse outcomes [3].

One of the most common clinical manifestations of RV is hand ischemia. Although not common, this presentation presents profound implications for patients with a prevalence of 0.5 % [4]. Beyond debilitating pain and compromised function, ischemic hands signal advanced vasculitis and the risk of significant tissue loss [5]. Such severe presentations require prompt and aggressive therapeutic interventions to prevent further disease progression, tissue necrosis, and potential loss of limb function.

In this context, we describe our experience with an elderly patient with RA who presented with complaints of polyarticular joint pain, a relatively routine presentation in our rheumatology clinic-exhibited ischemic hands. Clinical and laboratory evaluation revealed that RV was the underlying cause. Guided by evidence-based protocols and the patient’s clinical scenario, we opted for rituximab, an anti-cluster-of-differentiate-20 (anti-CD20) monoclonal antibody, which has shown efficacy in the management of RV [5].

Our case provides insights into the clinical journey of RV diagnosis and treatment and emphasizes the importance of being vigilant about signs, such as ischemic hands, in patients with RA. Early detection of these signs can drastically alter the clinical course, optimize the chances of effective treatment, and halt RV progression [6]. Thus, this study aimed to highlight the multifaceted considerations in managing such challenging scenarios and the importance of a nuanced approach to RA complications.

## Case presentation

An 83-year-old man presented to a rural community hospital with the chief complaint of systemic pain, mainly in the back and neck. He was initially independent in activities of daily living. Approximately 14 days before the visit, the patient experienced back pain. The pain increased on the day of his visit, and he was unable to walk, resulting in a visit to the hospital’s emergency department. His medical history included RA, diagnosed one year ago and treated by the rural community hospital's general physician. His medications included prednisolone at 5 mg/day and methotrexate at 12 mg/week for RA. 

The vital signs at the visit were as follows: blood pressure, 125/80 mmHg; pulse rate, 84 beats/min; body temperature, 36.8°C; respiratory rate, 14 breaths/minute; and oxygen saturation, 96% on room air. The patient was alert to time, place, and person. Physical examination revealed swelling and heat at the shoulder, wrist, and knee joints. Additionally, back and neck tenderness was observed in the supine position. No other neurological abnormalities were noted. No apparent abnormalities were found on the chest, abdomen, or skin. Laboratory tests showed elevated inflammation with hypocomplementemia (Table 1).

**Table 1:** Initial laboratory data of the patient eGFR, estimated glomerular filtration rate; CK, creatine kinase; CRP, C-reactive protein; C3, complement component 3; C4, complement component 4; MPO-ANCA, myeloperoxidase antineutrophil cytoplasmic antibody

Parameter	Level	Reference
White blood cells	7.5	3.5–9.1 × 10^3^/μL
Neutrophils	86.2	44.0–72.0%
Lymphocytes	10.0	18.0–59.0%
Monocytes	1.4	0.0–12.0%
Eosinophils	1.8	0.0–10.0%
Basophils	0.6	0.0–3.0%
Red blood cells	3.70	3.76–5.50 × 10^6^/μL
Hemoglobin	11.3	11.3–15.2 g/dL
Hematocrit	34.0	33.4–44.9%
Mean corpuscular volume	91.8	79.0–100.0 fl
Platelets	20.8	13.0–36.9 × 10^4^/μL
Total protein	6.2	6.5–8.3 g/dL
Albumin	2.4	3.8–5.3 g/dL
Total bilirubin	0.7	0.2–1.2 mg/dL
Aspartate aminotransferase	13	8–38 IU/L
Alanine aminotransferase	7	4–43 IU/L
Alkaline phosphatase	69	106–322 U/L
γ-Glutamyl transpeptidase	24	<48 IU/L
Lactate dehydrogenase	198	121–245 U/L
Blood urea nitrogen	33.6	8–20 mg/dL
Creatinine	0.62	0.40–1.10 mg/dL
eGFR	90.0	>60.0 mL/min/L
Serum Na	138	135–150 mEq/L
Serum K	3.9	3.5–5.3 mEq/L
Serum Cl	102	98–110 mEq/L
CRP	14.19	<0.30 mg/dL
Anti-nuclear antibody	40	<40
C3	74	86–164 mg/dl
C4	6	17–45 mg/dl
MPO-ANCA	<1.0	<3.5 U/ml
Urine test		
Leukocyte	Negative	Negative
Nitrite	Negative	Negative
Protein	Negative	Negative
Glucose	Negative	Negative
Bilirubin	Negative	Negative
Blood	Negative	Negative
pH	7.0	
Specific gravity	1.013	

Chest X-ray revealed no infiltration of the lungs. Head-to-pelvis CT was performed to systematically rule out abscess formation and lymphadenopathy, revealing no mass, pleural effusion, or ascites.

**Figure 1:**
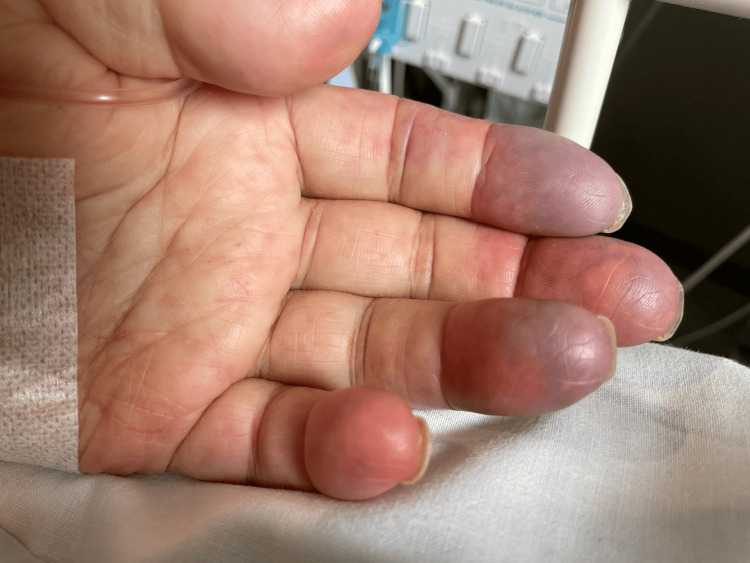
The ischemic fingers of the left hand

Based on the clinical findings of the worsening of polyarthritis and high inflammatory conditions, the patient was diagnosed with exacerbated RA. The patient was treated with an increased dose of prednisolone of 15mg. The patient’s symptoms were alleviated seven days later, and etanercept was administered at 50 mg/week. On admission day seven, the patient had a fever of 39°C and ischemic fingers on both hands (Figure 1).

The respiratory rate increased to 24 breaths/minute, and unconsciousness progressed. Sputum and urine samples did not show any signs of infection. Arterial ultrasound and contrast-enhanced CT revealed no occlusion of the arteries of the extremities. Clinically, he was suspected of sepsis without obvious infection origin and was treated with tazobactam and piperacillin for hospital-acquired infections. However, his fever persisted for one week, and blood, sputum, and urine cultures were negative. Further, his joint pain and swelling were exacerbated, and initial laboratory tests of the complement system showed a significant decrease (complement component 3/complement component 4 (C3/C4)) (Table 1). Ischemic changes in both his hands persisted. Based on the clinical worsening of rheumatic and systemic symptoms accompanied by hypocomplementemia without infectious causes, the patient was diagnosed with exacerbation of RV.

On admission day 10, the patient was started on methylprednisolone at 1000 mg/day for three days, followed by prednisolone at 60 mg/daily. After starting treatment, his joint pain improved, and the change in the ischemic hands disappeared, accompanied by the disappearance of fever. While he was rehabilitated to go back to his home, on admission day 22, he had a fever >39°C and polyarticular pains and swelling after prednisolone was reduced to 30 mg/day and ischemic hands reappeared. After ruling out bacteremia and other infections, such as lung and urine cultures for bacteria and fungus, and negative serologies of hepatitis B/C, on admission day 25, intravenous rituximab at 500 mg biweekly was administered to treat the exacerbation of RV. On admission day 29, his fever and polyarticular pain were mitigated, and rehabilitation was restarted. On admission day 37, he was transferred to a rehabilitation facility to prepare for discharge.

## Discussion

This case report describes an older patient with RA who began to show worsening symptoms. Further examination revealed changes in the hands indicative of vasculitis. RV was diagnosed, and timely intervention was administered. The patient was managed in a community hospital, and the adopted methods proved effective in treating RV.

General physicians should consider the possibility of RV in older patients with RA when symptoms exacerbate. General physicians should be vigilant about the onset or exacerbation of symptoms in older patients [7-9]. This may not just be a progression or flare-up of RA but could also indicate the development of RV. This distinction is critical because treatment modalities and prognoses differ. This learning point further emphasizes the importance of a detailed patient history and clinical examination to detect subtle changes or worsening in patients with RA [5,10].

This case reports the effectiveness of detecting changes in hands in determining vasculitis manifestations to treat RV effectively in community hospitals. Changes in the hands, such as nodules, skin ulcers, or Raynaud’s phenomenon, could be early and specific signs of RV in RA patients [3,11]. Early detection of these changes can significantly improve the management and prognosis of RV [4]. Community hospitals, which may not have the advanced imaging or laboratory facilities found in tertiary centers, can still play a pivotal role in diagnosing RV by paying keen attention to clinical manifestations, especially in the hands [12].

General physicians’ practical approaches to RV in community hospitals should be emphasized. General physicians in community hospitals are often the first line of defense against various diseases, including manifestations of RV [13]. RV can also show various skin lesions, such as palpable purpura, eczema, and erythema nodosum [13]. This case underscores the importance of continuous medical education and awareness among general physicians regarding the possible manifestations and complications of common diseases such as RA [14]. Early detection and referral, where required, can be life-saving for patients with RV [15]. Additionally, general physicians should be familiar with RV's management and treatment options as system-specific specialists, considering the resources available in a community hospital setting [16]. This case report serves as a valuable learning tool for clinicians, emphasizing the importance of thorough clinical examinations, especially in older patients with RA, and the role of community hospitals in managing diseases such as RV.

## Conclusions

This case report highlights the need for increased clinical awareness in older patients with RA, especially when exacerbated symptoms or changes in the hands indicative of RV are noted. Such observations are particularly essential for community hospitals as they may serve as initial contact points and may not possess advanced diagnostic equipment. General physicians play a vital role in timely and effective RV management through clinical observation and ongoing medical education.
